# Fibroblast-associated tumour microenvironment induces vascular structure-networked tumouroid

**DOI:** 10.1038/s41598-018-20886-0

**Published:** 2018-02-05

**Authors:** Sang Woo Lee, Hyeong Seob Kwak, Myoung-Hee Kang, Yun-Yong Park, Gi Seok Jeong

**Affiliations:** 1 Biomedical Engineering Research Center, Asan Medical Center, Seoul, Korea; 2 Asan Institute for Life Sciences, Asan Medical Center, Seoul, Korea; 3 Department of Convergence Medicine, University of Ulsan College of Medicine, Seoul, Korea

## Abstract

*In vitro* three-dimensional (3D) tumour models mimic natural cancer tissue *in vivo*, bridging the gap between conventional 2D *in vitro* testing and animal models. Stromal and cancer tissues with extracellular matrix (ECM) can provide a tumour microenvironment (TME) with cell-to-cell and cell-to-ECM interactions. These interactions induce the exchange of biophysical factors, contributing to the progression, metastasis, and drug resistance of cancer. Here, we describe a 3D *in vitro* lung cancer model cultured in a microfluidic channel that is able to confirm the role and function of various stromal cells in tumourigenesis, thereby representing an *in vivo*-like TME. We founded that biophysical factors contribute to the role of fibroblast cells in tumour formation, especially, producing a nascent vessel-like tubular structure, resulting in the formation of vascularized tumour tissue. Fibroblast cells altered the gene expression of the cancer cells to enhance metastasis, survival, and angiogenesis. The device could be used for developing and screening anti-cancer drugs through the formation of the same multicellular tumour spheroids under TME interactions. We believe this microfluidic system provides interaction of TME for cancer research by culturing stromal tissue.

## Introduction

*In vitro* three-dimensional (3D) tumour models mimic *in vivo* natural cancer tissue. *In vitro* models of cancer have played important roles in research, ranging from their use in basic cancer research to their use in anti-cancer drug discovery^1^. For decades, conventional two-dimensional (2D) culturing platforms have been used in anti-cancer drug development, drug screening, cancer therapies, and other cancer research^2^. Although 2D systems are still actively used for such purposes, in monolayer culture, these systems lack the complex 3D cell-to-cell and cell-to-extracellular matrix (ECM) networks of cancer, which limits their usefulness and can lead to misleading or unexpected results^3^. The 3D tumour model is, therefore, widely considered to fill the gap between conventional 2D *in vitro* testing and animal models^4–6^.

Cancer tissue that includes stromal cells and ECM can provide a tumour microenvironment (TME), *in vivo* and *in vitro*, in which cell-to-cell and cell-to-ECM interactions occur^2^. Through these interactions, cancer and stromal tissue induce the exchange of various biophysical and biochemical factors that contribute to the progression^7^, metastasis^8^, and drug resistance of cancer^1,9–13^. In particular, cancer-associated fibroblasts (CAFs) regulate the biophysical and biochemical factors of the TME that contribute to promoting the initiation and development of tumour formation^14,15^. Thus, a 3D *in vitro* tumour model with integrated ECM and stromal cells can play an important role, reflecting the *in vivo* TME^16–19^.

There is much published research regarding ways to improve both *in vivo* and *in vitro* strategies for developing *in vitro* tumour models^20–23^. One approach has been the creation of 3D multicellular tumour spheroids (MCTSs) with physiological characteristics similar to tumour tissue, replicating the *in vivo* TME^24^. Although these characteristics provide an *in vivo*-like environment similar to that of natural tumour tissue, there is still a considerable need for *in vitro* studies of tumour formation and growth processes in 3D tumour tissue for a range of applications, from basic studies to the screening of potential anti-cancer agents. As a result, there is growing interest in the development of a well-organized *in vitro* tumour model in which the long-term effects of anti-cancer drugs can be assessed^3,9,25^, in which the metabolic environment is similar to the natural tumour tissue environment and can be managed in real time^3^, and in which close interactions between cancer and stromal tissue within the TME can be maintained^17,19,26,27^.

In this study, we describe a 3D *in vitro* lung cancer model where tumour cells were cultured in a microfluidic channel, which provided interactions of the TME. Microfluidic technology that utilizes a variety of cells to model tumour microenvironment was reported in recent reviews^28,29^. Microfluidic channels allowed stable cell growth by providing a vessel-like channel through which there was a continuous flow of culture medium supplying oxygen and nutrients^30–32^. To construct this *in vitro* lung cancer model, endothelial cells, fibroblasts, and lung cancer cells were sequentially seeded and tri-cultured within a 3D collagen matrix. The non-small cell lung cancer (NSCLC) cell line A549 was considered suitable for identifying lung cancer heterogeneity^3^ and for studying NSCLC in a 3D lung TME^4^. The adenocarcinoma cell line (A549) have been commonly used by many researchers to study the cancer research via three-dimensional tumour spheroid formation^33–35^. To maintain an *in vivo*-like TME, the culture medium was continuously supplied through the vessel-like channel using a passive micropump. We found that the transforming growth factor β (TGFβ) and matrix metalloproteinase (MMP) released from the fibroblasts contributed to tumour formation in the microfluidic channel. Furthermore, the biophysical force exerted by the fibroblasts induced the formation of tumours and the vascular networking between neighboring *in vitro* tumours. Using mRNA analysis, a fibroblast co-culture model was shown to induce the upregulation of genes associated with metastasis and angiogenesis, as well as the downregulation of genes involved in apoptosis. To evaluate the drug response of the 3D tumour model, paclitaxel and gemcitabine (anti-cancer agents for NSCLC) were directly applied to the microfluidic channel.

## Results

### Production of an *in vivo*-simulating TME for *in vitro* 3D tumouroid formation

**Figure 1:**
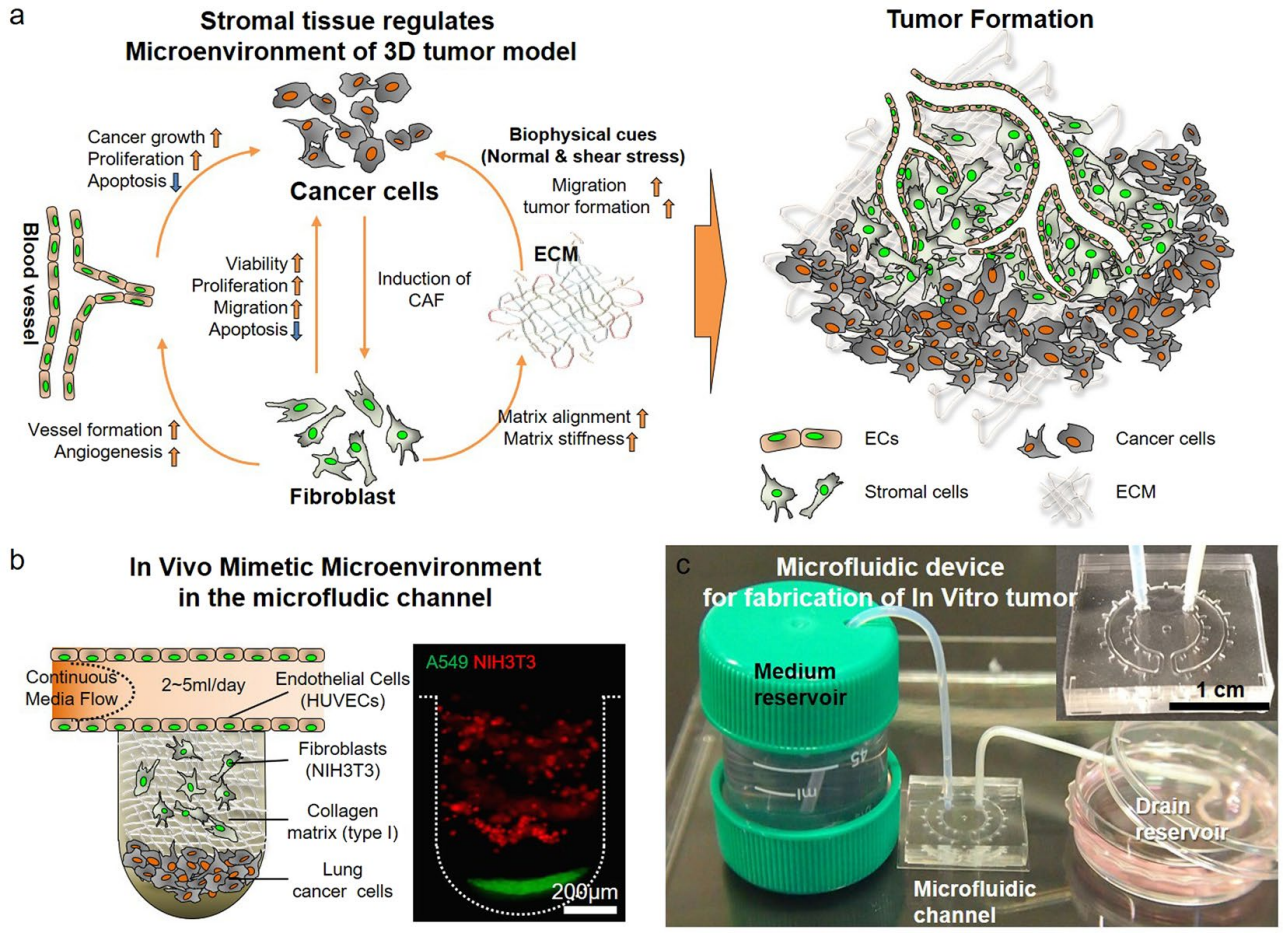
*In vivo* tumour microenvironment and *in vitro* tumourigenesis model. **(a)** Biophysical cues affected not only tumourigenesis but also tumour angiogenesis. Biochemical cues, such as cytokines and growth factors, promoted the induction of resident fibroblasts into cancer-associated fibroblasts (CAFs). CAFs in contact with cancer cells enhanced the viability, proliferation, and migration of these cells and reduced apoptosis. Fibroblasts in contact with the extracellular matrix (ECM) caused matrix alignment for tumour formation. Interactions between cancer cells and fibroblasts played a critical role in tumourigenesis through various physiochemical cues. **(b)** The microfluidic device was designed to supply the *in vitro* tumour microenvironment with oxygen and nutrients. Cancer and fibroblast cells were differentiated using a spin-down technique and confirmed with two fluorescence cell trackers. The continuous flow of the culture medium was regulated by yarn flow resistance inside the drain tube to maintain conditions similar to those of blood flow in a capillary.

Tumours have a complex architecture that consists of cancer and stromal tissue with a vascular structure surrounded by ECM (Fig. 1a). Close interactions between these elements play key roles in maintaining the TME^8^. These biophysical and biochemical interactions within the TME affect the progression, growth, and survival of the tumour^8,36^. To create an *in vivo-*simulating representation of the TME, we designed a microfluidic device that integrated cancer and stromal cells, fibroblasts, and endothelial cells, which were surrounded by a 3D collagen matrix with a channel for the continuous flow of culture medium (Fig. 1b). To fabricate the microfluidic channel, PDMS mold, and cover glass were performed oxygen plasma treatment together, and bonded (Supplementary Fig. 1a). Lung cancer cells (A549, green) and fibroblasts (NIH3T3, red) were sequentially seeded into microwells (Fig. 1b, and Supplementary Fig. 1b) to form the *in vivo*-simulating TME in the microfluidic channel. We verified the density of cells, which plated within microwells by microspin-down technique, via fluorescence intensity average. It was confirmed that the density of cells applied to each microwell showed a similar amount (Supplementary Fig. 2). The flow of the cell culture medium through the channel was supported by a siphon effect driven by a passive micropump so that oxygen and nutrients were continuously provided to the TME. The flow rate was maintained at approximately 2–5 ml/day using cotton yarn resistance in the tube^37^. Through these means, it was a suitable tool to confirm the interaction of TME between cancer and stromal tissue in the microfluidic devices (Fig. 1c).

### Verification of the promotion of tumourigenesis by tumour-resident fibroblasts

To demonstrate the *in vivo* like TME, we first confirmed the expression of CAF-specific markers in the NIH3T3 cells to demonstrate the role of these cells as a function of tumour-resident fibroblasts, such as CAFs, during the formation of the *in vitro* tumour. When treated with TGFβ, NIH3T3 fibroblasts express a CAF-specific marker^38^. During tumouroid formation, TGFβ was highly expressed throughout the entire area of the tumouroid, including the collagen matrix. Of the epithelial-mesenchymal transition (EMT) markers, vimentin for the mesenchymal lineage was highly expressed in NIH3T3, whereas E-cadherin (for the epithelial lineage) was less strongly expressed. The expression of αSMA, FAP, and PDGFRβ (typical specific positive markers of CAFs) was confirmed in cultivated NIH3T3 cells. The expression of the proliferation marker Ki-67 was also high. Cytokeratin and CD31, which are negative markers, were not expressed (Supplementary Fig. 3). The characterization of fibroblasts was distinguished through comparison to the expression of CAF markers in the A549 lung cancer cells (Supplementary Fig. 4). Thus, we verified that NIH3T3 cells in the *in vitro* TME can serve as CAFs by developing distinct cellular characteristics via the expression of CAF-specific markers.

### Fibroblast-associated microenvironment regulating the *in vitro* tumouroid formation

**Figure 2:**
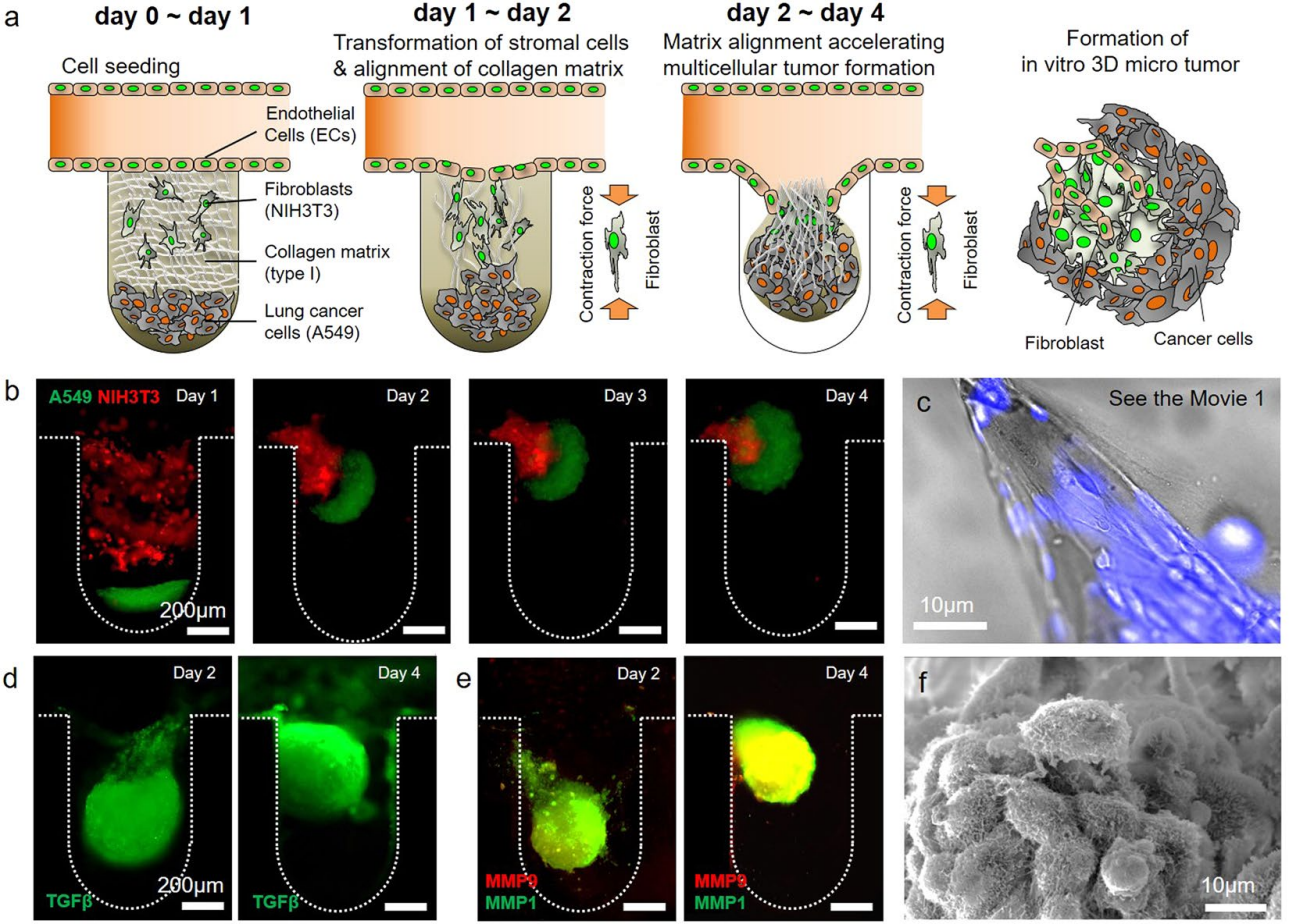
Fibroblast-associated microenvironment during formation of an *in vitro* tumouroid. **(a,b)** Cellular constituents and collagen type I matrix of the *in vitro* tumour microenvironment developed a spherical structure of cancer cells in the microwell of the microfluidic device. On days 1 and 2, the collagen matrix was aligned by contraction forces due to the transformation of fibroblasts on the collagen matrix (see Movie 1). Fibroblasts attached to the aligned collagen matrix during tumour formation could be distinguished from cancer cells. During days 2–4, multicellular tumour spheroids (MCTSs) formed rapidly with the alignment of the collagen matrix. The morphology of these MCTSs persisted during tumourigenesis. Microvilli for cell-to-cell interactions of cellular constituents were activated on the MCTSs. **(c)** Alignment of the collagen matrix by fibroblasts in the microwell was observed during tumouroid formation. **(d)** TGFβ was highly expressed during tumour formation under the condition of fibroblasts. **(e)** During tumour formation, MMP1 was expressed on fibroblasts attached to the collagen matrix, and MMP9 was expressed on aggregated cancer cells. **(f)** Cell-to-cell interactions were observed in activated microvilli rather than in fibroblast-free MCTSs in the tumour formed by fibroblasts.

The process of *in vitro* tumouroid formation in the microfluidic device is depicted in Fig. 2a. After creating the *in vitro* TME, the collagen matrix and cells became detached from the bottom line of the microwell on day 1 (Fig. 2b, Day 1). On days 2–4, cancer and stromal cells, fibroblasts, and human umbilical vein endothelial cells (HUVECs) aggregated and formed a tumouroid (Fig. 2b, Days 2–4). During this process, fibroblasts exerted a force on the collagen matrix that contributed to the alignment of the matrix and migration of the tumouroid (Fig. 2b and Supplementary Movie 1). Cancer and stromal cells with the collagen matrix spontaneously aggregated and formed a complete spheroidal tumouroid on days 2–4 and migrated into the culture medium channel (Fig. 2b and Supplementary Fig. 5). Figure 2c shows the aligned collagen matrix resulting from the contraction force of the fibroblasts. During this process, cancer and fibroblast cells were not mixed but aggregated separately in different areas (Fig. 2b and Supplementary Fig. 5). The HUVECs in the culture medium channel also migrated and aggregated with the *in vitro* tumouroid (Supplementary Fig. 6).

There is a close interaction between the local biochemical microenvironment and tumour formation and cancer growth^39^. We demonstrated this biochemical environment interaction during the tumour formation (Fig. 2d). At the early stage of tumouroid formation, TGFβ was expressed throughout the entire tumouroid area of (Fig. 2d, Day 2). Although TGFβ was expressed in culture models with and without fibroblasts, it was highly expressed across the whole area of the tumouroid with the fibroblast culture model (Supplementary Fig. 7a). Without fibroblasts, it was only expressed at the edge of the collagen matrix and HUVECs during the early stage of the process (Supplementary Fig. 7a). We confirmed that MMP1 and MMP9 were highly expressed in both culture models (Fig. 2e and Supplementary Fig. 7b). Although these MMPs were highly expressed at the early and late stages, there was no spheroidal formation in the culture model without fibroblasts. MMP 1 and MMP9 were expressed in different areas (Supplementary Fig. 7b). However, with the fibroblast culture model, the expression of MMPs was relatively evenly distributed throughout the tumouroid (Fig. 2e and Supplementary Fig. 7b). Cell-to-cell interactions were observed in activated microvilli rather than in the tumour spheroids under the condition without fibroblasts in the tumour rapidly formed by fibroblasts (Fig. 2f, Supplementary Fig. 8).

### Vessel-like tubular structure formation between tumouroids in the microfluidic channel

**Figure 3:**
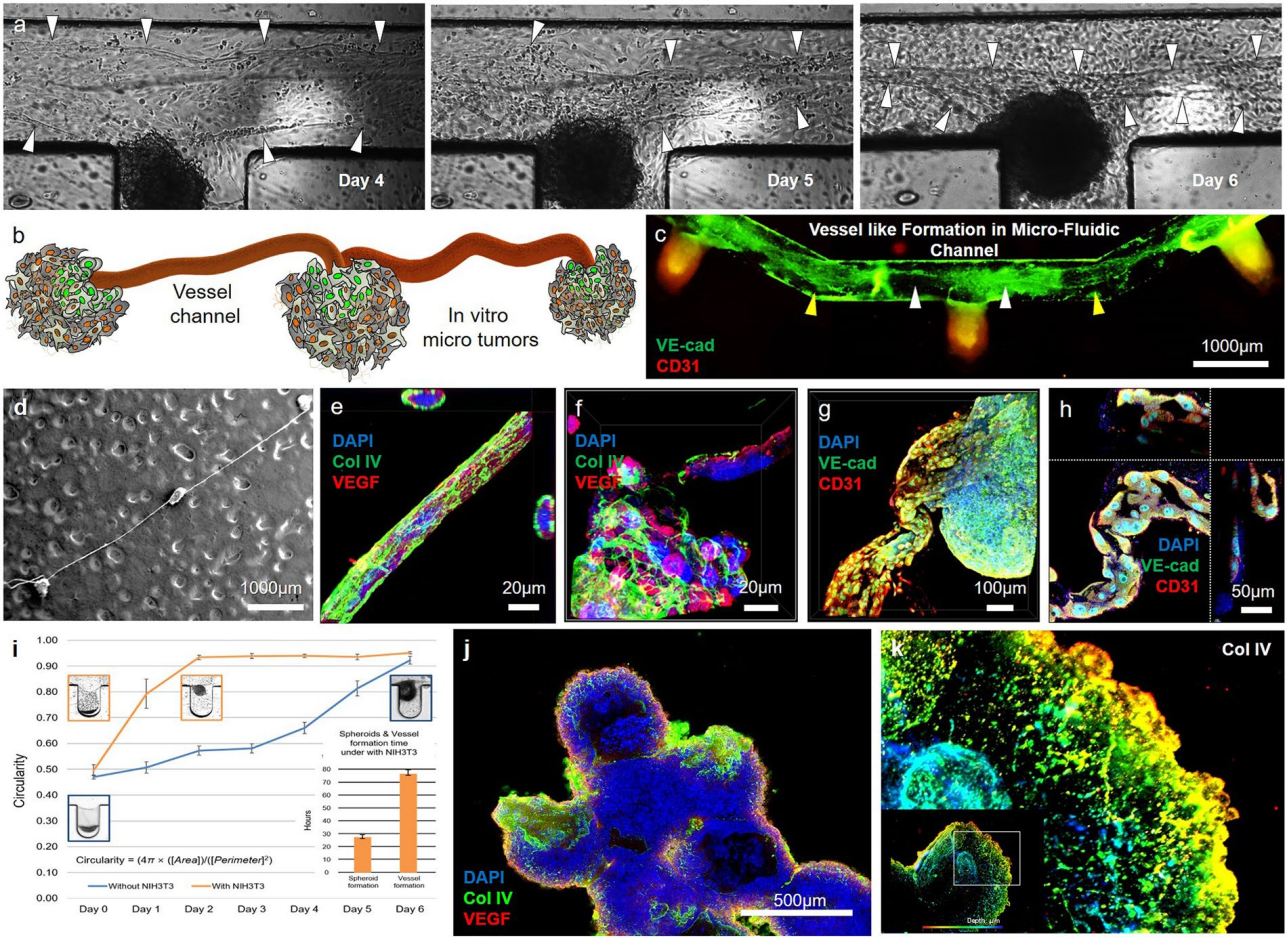
Vessel-like tubular structure formation in the microfluidic device. **(a)** The collagen matrix was stretched by tension forces between neighboring MCTSs due to fibroblasts. After stretching, the collagen matrix sheet was rolled up by a stretching force between the MCTSs. **(b)** The collagen matrix stretched by the MCTS fibroblasts exhibited torsion between neighboring MCTSs. **(c)** Stretching (yellow arrow heads) and torsion (white arrow heads) of the collagen matrix were revealed on a collagen matrix sheet stained with blood vessel-related markers (VE-cad, CD31). **(d)** The vessel structure connecting the MCTSs was tight and stable. **(e)** The collagen IV matrix formed a net structure and enclosed HUVECs. **(f)** The collagen IV matrix was also tightly formed at the site of the MCTS fibroblasts. **(g,h)** VE-cad and CD31 were expressed only on a vessel-like tubular structure distinct from the MCTS. This distinct tissue formed the lumen composed of HUVECs. **(i)** The spheroid morphology (with circularity ≥ 0.9) formed more quickly MCTSs with NIH3T3 (orange curve and box) than without NIH3T3 (blue curve and box). The vessel-like tubular structure was formed within 80 h after the formation of spheroids (orange bar) under the condition with NIH3T3 (purple bar) but not without. **(j)** The MCTSs formed a large tumour mass. **(k)** In the 3D image of the MCTS, collagen IV was expressed in a linear shape similar to that of a capillary vessel, from the outer layer of the spheroid to the interior space (colors indicate the depth of the tumouroid).

After tumouroid formation, we observed vessel-like tubular structures generated between the tumouroids in the culture medium channel. A sheet of HUVECs in the channel was stretched between neighboring tumouroids. On days 4–6, the HUVEC sheet between the tumouroids stretched continuously, resulting in it rolling up and developing a vessel-like tubular structure (Fig. 3a and Supplementary Movie 2). Figure 3b and c show a schematic and a fluorescent image of this structure, where networked (VE-cad expressed region) tumouroids formed in the channel. Figure 3c shows a fluorescence microscopic image of the vessel-related markers VE-cad and CD31 in the channel. Scanning electron microscope (SEM) images revealed the presence of these vessel-like tubular structures, connecting the microtumours 10 days after seeding the cells and the collagen matrix (Fig. 3d). The structures were tightly connected to the type IV collagen mesh, and the cross-sectional view clearly shows their tubular structure by NIH3T3 fibroblasts (Fig. 3e). In addition, the microtumours were robustly developed and surrounded by a type IV collagen matrix (Fig. 3f). The tubular structure composed of the collagen IV matrix and HUVECs showed expression of the endothelial cell markers VE-cad and CD31 and was tightly connected to the microtumours in a shape similar to that of a blood vessel (Fig. 3g and h). Figure 3h shows a magnified confocal microscopic image of the vessel-like structure, within also had an internal hollow structure. The vessel-like tubular structure formed after the rapid formation of the tumour spheroid (Supplementary Movie 1 and 2) through the biophysical effect of NIH3T3 cells in the TME. Figure 3i describes this formation period. The spheroidal microtumours formed rapidly within 24 h of co-culturing with fibroblasts; the vessel-like tubular structure then developed over the course of approximately 75 h. However, without fibroblasts, the vessel-like tubular structure did not develop in our microfluidic device. The tumour spheroids that formed in the device were harvested and subjected to suspension cultivation. All spheroids were connected by a vessel-like tubular structure and exhibited microvessel formation. Finally, a large tumour mass approximately 2 mm in size was produced within 2 weeks using the microfluidic system (Fig. 3j). Type IV collagen was expressed in a linear shape similar to that of a capillary vessel, from the outer layer of the tumour spheroid to the interior space, as observed using depth coding analysis of the 3D image (Fig. 3k).

### Gene expression profile from the microfluidic model simulating tumour growth

**Figure 4:**
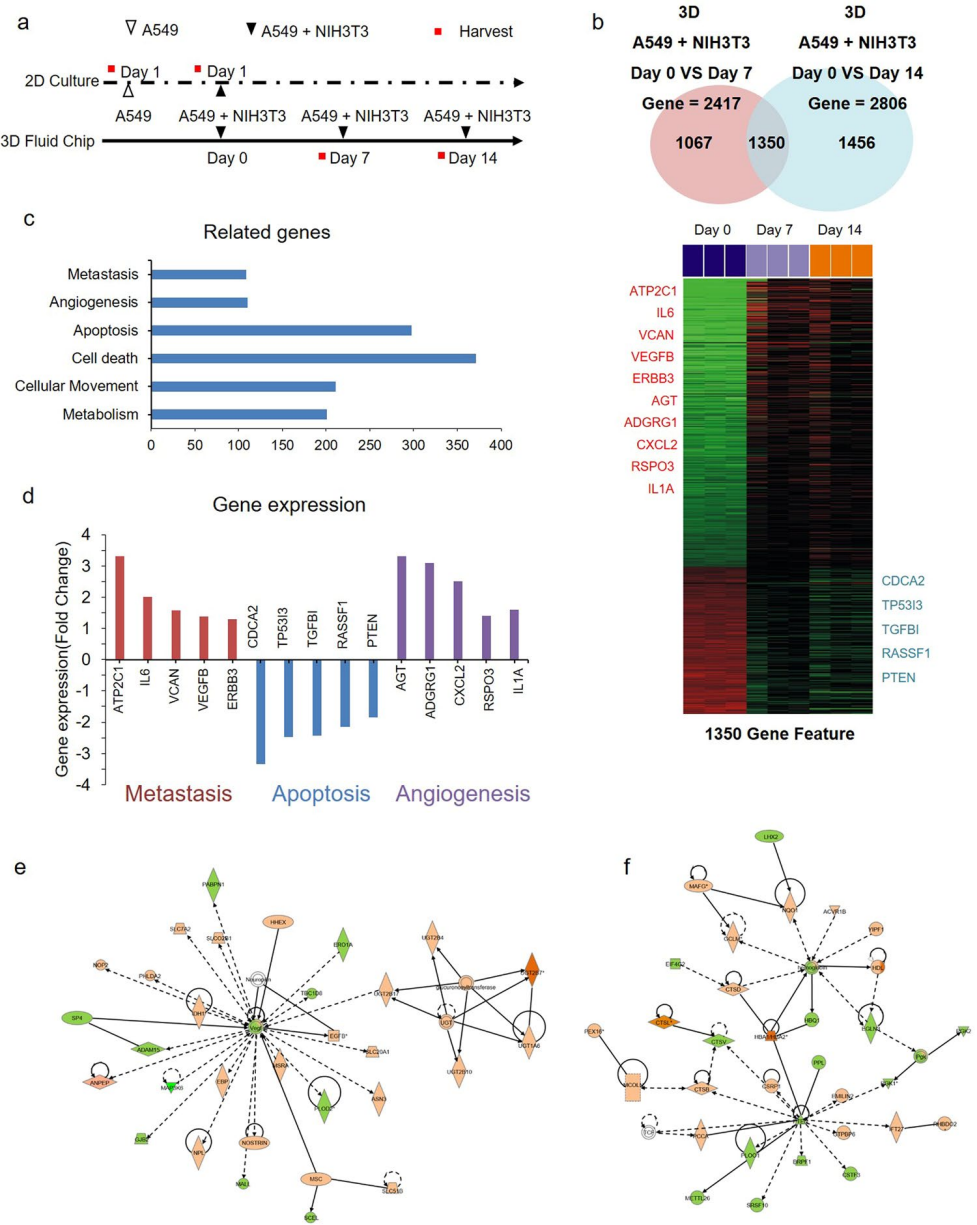
Change in gene expression by fibroblasts during tumour progression. **(a)** Experimental scheme for gene expression profile. Indicated cells were cultured in 2D and in the 3D microfluidic device, and RNA was extracted from cells at the indicated time points. **(b)** RNA was used for the microarray to establish the gene expression profile. The Venn diagram presents genes expressed differentially between groups of arrays; the expression of 1350 genes was visualized using Heatmap software. **(c)** Total of 1350 genes was analyzed based on Ingenuity Pathway Analysis (IPA), and the signaling pathway was categorized according to IPA results. **(d)** Expression levels of genes involved in metastasis, apoptosis, and angiogenesis are shown. **(e,f)** For gene networks, VEGF and PTEN-enriched signaling pathways were visualized based on IPA analysis. Abbreviations: ATP2C1; Calcium-transporting ATPase type 2C member 1, IL6; Interleukin-6, VCAN; Versican, VEGFB; Vascular endothelial growth factor beta, ERBB3; Erb-B2 receptor tyrosine kinase 3, CDCA2; Cell division cycle-associated protein 2, TP53I3; Tumour protein p53 inducible protein 3, TGFBI; Transforming growth factor beta induced, RASSF1; Ras Association Domain Family Member 1, PTEN; Phosphatase and tensin homolog, AGT; Angiotensinogen, ADGRG1; Adhesion G protein-coupled receptor G1, CXCL2; Chemokine ligand 2, RSPO3; R-spondin 3, IL1A; Interleukin 1 alpha.

To evaluate the molecular features of the *in vitro* tumouroid model between with- and without fibroblasts, we generated gene expression profiles, as shown in Fig. 4a. Using the BRB array tool of the Biometric Research Program, we selected genes that were significantly expressed between samples (Fig. 4b). We subjected the differentially expressed genes to pathway analysis using Ingenuity Pathway Analysis (IPA). As expected, the IPA results revealed enrichment of the gene ontology associated with tumour metastasis, angiogenesis, and cellular movement (Fig. 4c). For metastasis, apoptosis, and the angiogenesis pathway, we listed genes involved in the signaling pathway (Fig. 4d). To observe how those genes built a network within the cancer-associated genes, we generated a gene network based on the IPA analysis. VEGF was networked with cancer-associated genes (Fig. 4e); the well-known tumour suppressor PTEN, which is down-modulated in metastatic cell systems, was associated with a gene associated with cell proliferation (Fig. 4f), suggesting that our metastasis model reflected well the physiological conditions and biological metastatic property. Taken together, the gene expression profile data suggested that our model could feasibly be used to examine metastatic features correlated with biological phenomena *in vitro*.

### Anti-cancer drug screening evaluation for the tumouroid

**Figure 5:**
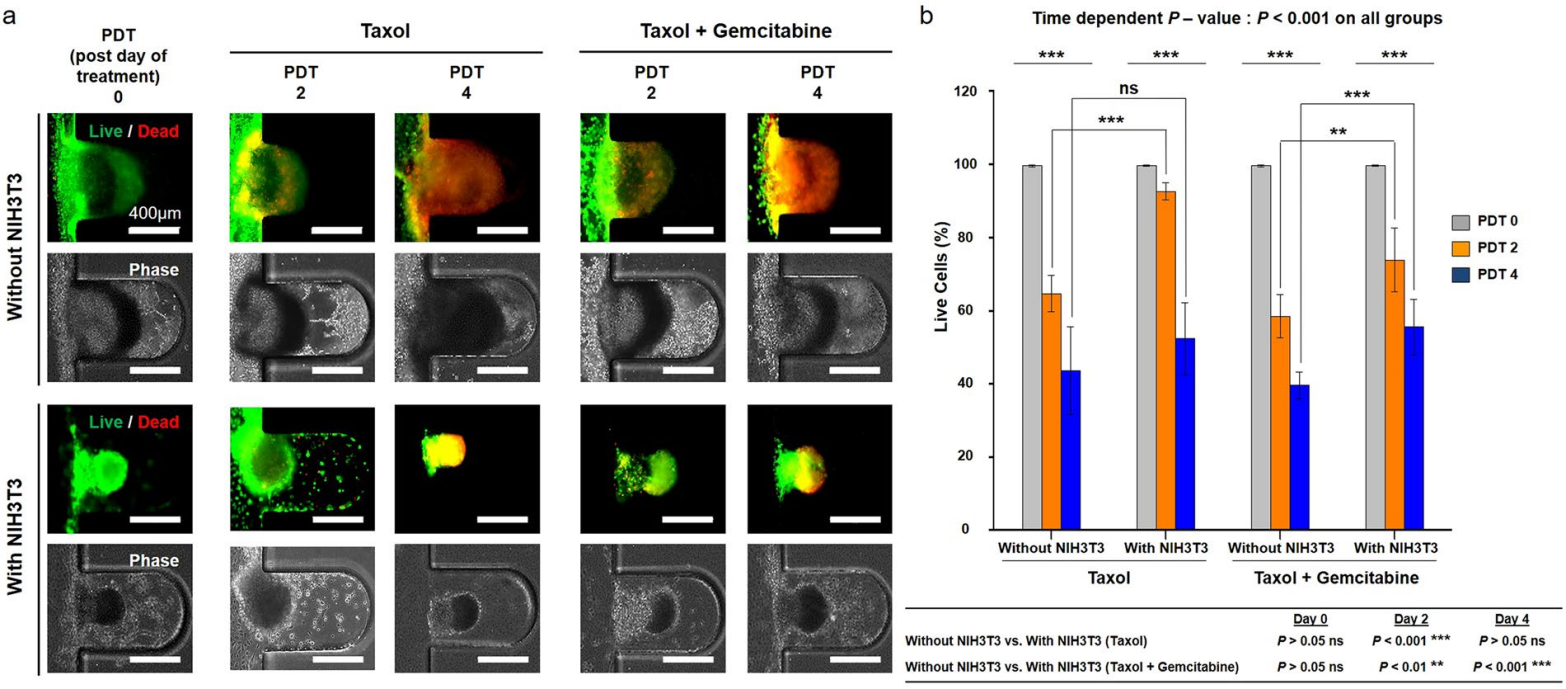
Screening of anti-cancer drugs on multicellular tumour spheroids (MCTSs) without and with fibroblasts. **(a)** Live and dead cells (green represents calcein-AM staining, indicating live cells, and red represents ethidium homodimer staining, indicating dead cells) after treatment of MCTSs with paclitaxel (Taxol) or a drug combination (paclitaxel + gemcitabine), administered as part of the medium flowing through the microfluidic device, without and with NIH3T3 fibroblasts. Both with- and without NIH3T3 fibroblasts, the tumour spheroid morphology remained after administration of paclitaxel or the drug combination. The proportion of live cells gradually decreased over 4 days of post-day of treatment (PDT). **(b)** After treatment with anti-cancer drugs, there was a gradual decrease in live cells in all groups, depending on the time period of drug treatment. The percentage of live cells was lower in combination treatment groups than in paclitaxel-only groups. The percentage of live cells was higher with fibroblasts than without. (**p < 0.01; ***p < 0.001).

To confirm the characteristics of the anti-cancer response of the microfluidic system, we treated the tumouroid with anti-cancer drugs administered through the culture medium channel. Tumour spheroids (n = 8) were selected and evaluated by fluorescence intensity average by live and dead staining within the microfluidic device. Figure 5a shows images of live and dead cells (green represents calcein-AM staining, indicating live cells, and red represents ethidium homodimer staining, indicating dead cells) after treatment with paclitaxel or a combination of paclitaxel + gemcitabine, without and with NIH3T3 fibroblasts. Both with- and without fibroblasts, the tumour spheroids retained their morphology after drug treatment. Over the four days following treatment with the anti-cancer drugs, the number of live cells gradually decreased in all groups, depending on the time period of drug treatment. The percentage of live cells was lower after combination treatment than after paclitaxel alone and was higher with fibroblasts than without (Fig. 5b). To support the influence of fibroblasts, comparative drug response study was also performed in 2D culture condition. The results showed that the drug resistance by NIH3T3 fibroblasts was higher through the expression of high live cell portion. (Supplementary Fig. 9)

## Discussion

### Fibroblast-associated tumour microenvironment regulating tumouroid formation in the microfluidic device

The TME is an ensemble of many cellular and structural constituents. Each constituent represents biochemical and biophysical cues for activation of tumourigenesis in cancer cells (Fig. 1a). In this study, we aimed to represent the *in vitro* custom-shaped vascularized heterotypic tumour tissue within the microfluidic device. It has been reported that CAFs are a major constituent of the tumour stroma by many researchers^40–43^. CAFs commonly display myofibroblastic characteristics, including a prominent contractile ability and expression of αSMA. CAFs play key roles in promoting cancer progression by modification of extracellular matrix components. Although, NIH3T3 cell line is originated from a mouse embryo it was reported that can play a role of CAFs in studies^44–47^. Saito *et al*. explained that the secreting factors from NIH3T3 promoted the tumour progression of A549 human lung cancer cells^45^. Then, some researchers produced heterotypic 3D tumour models with human cancer cells and NIH3T3 cells^46,47^. Based on these researches, we produced *in vitro* 3D tumour model using human lung cancer cell line A549 and mouse fibroblast NIH3T3 cells within the microfluidic device which simulated *in vivo* TME. In results, the presence of fibroblasts enhanced the formation of the *in vitro* tumour model; compared with the absence of fibroblasts, when fibroblasts were present, tumour-like structures formed rapidly (Supplementary Fig. 7 and Supplementary Movie 1). It is well known that biophysical effects due to *in vivo* TME resident fibroblasts increase the malignancy of cancer cells through ECM remodeling and increased stiffness via matrix alignment^48^. In particular, CAFs regulate the biophysical and biochemical properties of the TME; this regulation promotes the initiation and development of tumour formation^14,15^. Furthermore, with biochemical effects such as the secretion of various cytokines and growth factors, cancer cells exhibit increased viability, proliferation, and migration capability and decreased apoptosis^49,50^. The formation of *in vitro* tumouroids, identified by the degree of circularity of the spheroid, was quicker with fibroblasts (<2 days) than without (>5 days). In addition, high expression levels of TGFβ and MMPs at the aggregation point of the NIH3T3 cells and collagen matrix showed that TME-resident fibroblasts affected cancer cells via the storage of MMP1 and MMP9 in the ECM. MMP9 is reported to be involved in cleaving fibrillary type I collagen, to which endothelial cells are exposed in an injured tissue that allows growth factor-induced angiogenesis^51^. Moreover, MMP9 may enhance local angiogenesis by increasing bioavailable levels of VEGF^52^ as a growth factor that is critical for the survival and growth of endothelial cells^53^ due to its ability to cleave membrane-bound VEGF. At this time, the expression of TGFβ was higher in the cancer cells and ECM than in the fibroblast-free condition (Fig. 2d and Supplementary Fig. 7). These findings demonstrate the ability of the proposed method to model an appropriate tumourigenic biophysical environment within a microfluidic device and to simulate the process of tumour formation *in vivo* via cell-to-cell and cell-to-ECM interactions.

### Fibroblast-induced vasculogenesis in the *in vitro* TME

Several strategies have been used to achieve *in vivo*-like tumour formations with vascularized complex structures^54–58^. In these studies, the most established system used sprouting angiogenesis methods that include providing adequate ECM and co-culturing cancer cells with stromal cells. Unlike sprouting angiogenesis, vasculogenesis is *de novo* vessel formation via migration of endothelial progenitor cells. The process of vasculogenesis first involves the induction of hemangioblasts and angioblasts, followed by the assembly of vessels^59,60^. In this microfluidic device, the *in vitro* tumour model described here enabled long-term survival (>2 weeks) in the microfluidic channel using a passive micropump system. This maintained suitable conditions for the formation of a stable *in vitro* tumour model with a complex structure. HUVECs formed the sheet, for example as a quiescent endothelial cell monolayer in the microfluidic channel. Cell migration and alignment were induced by activation of TGFβ (Fig. 2d) as angiogenic stimuli^61^ for vascular morphogenesis by resident mesenchymal stromal cells in the microwells of the microfluidic *in vitro* TME. The tubular structure was developed by synthesis and matrix reorganization of collagen type IV as a basement protein by NIH3T3 cells in the *in vitro* TME. VE-cad expressed at an early stage of vascular tube formation^62^ was found on a tumouroid-connected vessel-like tubular structure in this system. Accordingly, this tubular structure was evaluated as an early stage of vascular tube formation. As a result, a maturing vessel-like tubular structure formed between neighboring tumours in the microfluidic device (Fig. 3a–h). Furthermore, the activation of cell-to-cell interactions by resident fibroblasts in the TME resulted in the formation of a large-scale tumour mass due to the tight interaction of the fibroblast area with tumour spheroids isolated from the microfluidic device.

### Genetic changes by TME-resident fibroblasts during tumourigenesis

We analyzed changes in gene expression through mRNA to identify the cellular activation exchange of cancer cells by resident fibroblasts during tumourigenesis. The gene expression changes showed significant differences in 1,350 genes altered by NIH3T3 fibroblasts, as the resident fibroblasts of cancer cells (Fig. 4a,b). The genes related to apoptosis, cell death, and angiogenesis, which affect the proliferation and growth of cancer cells, significantly changed. These changes were confirmed to indicate evasion of growth suppressors, sustained proliferative signaling, activation of invasion, and metastasis of cancer cells through the influence of CAFs in the TME. These findings demonstrated that the microfluidic system described here could reflect changes in gene expression through long-term co-culture with stromal cells.

### Increased resistance to anti-cancer drugs by TME fibroblasts

Stromal fibroblasts in tumour cells could potentially contribute to drug resistance via various mechanisms such as the formation of a compact matrix structure and the creation of a physical barrier formed from stroma proteins that prevent intra-tumoural drug penetration^63^. As mentioned earlier, there is an increasing number of studies on the relationship between stromal cells, including CAFs, and drug resistance^64–66^. Here, the presence of fibroblasts in the *in vitro* TME led to the formation of a compact matrix (Fig. 5a), which did not occur when fibroblasts were absent, and compact forms of the tumour spheroids developed. Studies on drug screening test using 3D tumour models have been reported by many researchers^67–70^. However, the criteria for the drug treatment period was not clearly defined. Thus, we referred to the drug screening study using the microfluidic device reported by Patra *et al*. in 2016^68^. Meads *et al*. classified the role of CAFs in drug resistance as either cell adhesion-mediated drug resistance (CAM-DR) or soluble factor-mediated drug resistance (SFM-DR)^71^. After treatment with paclitaxel or a combination of paclitaxel + gemcitabine, the greater enlargement in the number of live cells in the tumour spheroids in the presence of fibroblasts than in their absence (Fig. 5a,b) supported the CAM-DR theory. This theory explains that increased drug resistance is due to a physical barrier. As an example of SFM-DR by CAFs, studies of the drug resistance of melanoma to chemotherapeutic agents have shown that CAFs assisted in metastasis and drug resistance by increasing the expression of IL-6, IL-8, MMP1, MMP2, and MMP9^72,73^. In particular, MMP1 and MMP9 level were increased in 3D lung model on a study published by *D*. Mishra *et al*. in 2012^74^. The enhancement of MMP1 and MMP9 was also observed a similar pattern in this study. The enhanced expression of MMP1 and MMP9 by resident fibroblasts of the TME (Fig. 2e and Supplementary Fig. 7b) can be explained as demonstrating drug resistance to chemotherapeutic agents through soluble factors. Thus, the findings of the present study showed that fibroblasts in the TME formed evolved tumour spheroids and increased drug resistance via various mechanisms, thereby reducing the therapeutic effect of anti-cancer drugs.

## Conclusion

The microfluidic device we produced was designed to provide *in vitro* tumouroids connected with vessel-like tubular structure. In this study, verifying the role of the applied fibroblasts in tumour formation confirmed the presence of a biophysical force; this force enabled the formation of a nascent vessel-like tubular structure, resulting in the formation of a vascularized tumour tissue. These results indicate the possibility of vasculogenesis via a novel method involving the biophysical factors of TME-resident fibroblasts, rather than by vessel-like formation by sprouting vascular endothelial cells and vessel matrix protein secretion. The microfluidic system provides interaction of TME for cancer research by culturing stromal tissue. The microfluidic device could be used for the development and screening of anti-cancer drugs by the formation of the same MCTSs under similar conditions. Authors consider this model will be able to a pre-animal model for cancer research with patient’s derived cells on further study.

## Materials and Methods

### Production of the microfluidic system

The poly(dimethylsiloxane) (PDMS; Sylgard^®^ 184, Dow Chemical Co., MI, USA) micro-channel was replicated from a master mold patterned with SU-8, which was made using conventional soft lithography on a silicon wafer (MicroChem, MA, USA). The mixture of a PDMS prepolymer containing a PDMS precursor and curing agent in a 10:1 ratio was decanted into the SU-8 mold and solidified in a drying oven (at 60 °C) for 2 h. The inlet and outlet holes were made using a punch (diameter, 2 mm). To generate the microfluidic system, the PDMS channel and cover glass were sterilized using an autoclave (JEIO TECH, Daejeon, Korea), and the replicated PDMS was bonded to the cover glass after treatment with oxygen plasma (Femto Science, Korea) (Supplementary Fig. 1a).

### Cell culture and generation of the *in vitro* tumour model

The A549 cell line (a human lung adenocarcinoma cell line, ATCC; CCL-185, Manassas, VA, USA) and the NIH3T3 cell line (a mouse fetal fibroblasts cell line, ATCC; CRL-1658, Manassas, VA, USA) were cultured in RPMI-1640 (Thermo Fisher Scientific, MA, USA) and Dulbecco’s modified Eagle’s medium (Thermo Fisher Scientific), with 10% fetal bovine serum (FBS, Biowest, MO, USA) and 1% antibiotics (Thermo Fisher Scientific). Human umbilical vein endothelial cells (HUVECs, ATCC; CRL-1730, Manassas, VA, USA) were grown in EGM2 (Lonza, Basel, Swiss) as the endothelial cell medium, consisting of 500 ml basal medium, 5% FBS, 20 ng/ml vascular endothelial growth factor (VEGF), and 1% antibiotics. To generate the *in vitro* tumour model, the microfluidic system was coated with 2 mg/ml of type I collagen solution (BD Bioscience, San Jose, CA, USA) using a micropipette. The pH of pre-polymerized collagen solutions was maintained naturally *in vivo* condition, ranging from 7.4 to 8.0. The A549 and NIH3T3 cells (2.0 × 10^6^ cells/ml) were prepared as suspensions in 2 mg/ml type I collagen solution. After removing the remaining collagen solution through aspiration, separate from the collagen filling the microwells, collagen premixed with the A549 cells was gently injected into the microfluidic system through the inlet hole, and the device was centrifuged at 6,000 revolutions per minute (RPM) for 30 s using a minicentrifuge (KA.MC-01; Korea Ace Scientific, Seoul, Korea) to trap the cancer cells in the microwell. After aspiration of the remaining cancer cells in the channel, the collagen solution premixed with NIH3T3 cells was seeded in a microfluidic system in the same way as for A549 cells. It was then centrifuged to exist over the cancer cell layer in the microwell. To remove the remaining cells and collagen solution, the microfluidic system was rinsed with a collagen injection and aspirated three times. It was then incubated at 37 °C for 30 min for the gelation of the collagen in the microwell. After this, HUVECs (1.0 × 10^6^ cells/ml) were seeded in a microfluidic channel to generate a vessel-like environment and incubated for 3 h at 37 °C so that they adhered to the vessel-like surface (Supplementary Fig. 1b). Following cell attachment, the microfluidic system was linked to the passive micropump established in our previous study^37^. The interactions among the cancer cells, fibroblasts, and HUVECs were monitored daily using a phase-contrast microscope (EVOS; Life Technologies, Carlsbad, CA, USA).

### Fabrication of a powerless micropump

A passive micropump driven by the siphon effect and controlled by yarn fiber resistance (YFR) was used to continuously supply the cell culture medium. Briefly, a cell culture medium reservoir was connected to the microfluidic channel via a polyethylene (PE) tube (optical density, 1.6 mm; internal diameter, 1.0 mm; Kinesis, Cambridge, UK), and cell culture medium waste from the device was drained into the outlet reservoir via a 10-cm-long PE tube filled with yarn fiber. After connecting the pump to the microfluidic cell culture system, the flow rate in the microchannel was maintained at approximately 2-5 ml/day.

### Fluorescence tracker labeling

The A549 cells, HUVECs, and NIH3T3 cells were fluorescently labeled using PKH67 (green; Sigma-Aldrich, St. Louis, MO, USA) or PKH26 (red; Sigma-Aldrich). The cells were placed in a conical tube at a cell density of 2 × 10^6^ cells and washed once using medium without serum. After centrifugation (400 *g*) for 5 min, the supernatant was carefully aspirated to leave 25 μl of supernatant. The cells were suspended by adding 150 μl of Diluent C solution to the pellet. Immediately, the dye solution was prepared with Diluent C, which was added with a PKH ethanolic dye solution (2 μl). The cells with Diluent C solution and the prepared dye solution were rapidly mixed and gently suspended. After incubation at room temperature for 5 min, an equal volume of a suitable protein solution (1% bovine serum albumin) was added to stop the staining, and the resulting solution was incubated for 1 min to allow binding of the excess dye. The solution with the stained cells was centrifuged at 400 *g* for 10 min at 20–25 °C and the supernatant was removed. The cell pellet was re-suspended in 10 ml of complete medium and transferred to a fresh sterile conical tube. Cells were centrifuged at 400 *g* for 5 min at 20–25 °C and washed twice with 100 ml complete medium to ensure the removal of any unbound dye. After the final wash, the fluorescent dye-stained cells were used in experiments to confirm their position and migration.

### Harvesting of MCTS and vessel-like tubular structure

After formation of MCTS and vessel-like tubular structure, they were harvested by introducing the culturing medium through the inlet of the microfluidic channel. For harvesting the connected MCTSs, the microfluidic chip was tapping gently, and pipetting was also gently conducted for a few times through the inlet of the chip. After introducing the culturing medium, finally, MCTSs connected with vessel-like tubular structure were harvested from the microfluidic channel. The vessel-like tubular structure connected MCTSs were conserved in the culturing medium to observe the morphology.

### Immunofluorescence assay

The *in vitro* MCTSs that formed in the microfluidic system were fixed with 4% paraformaldehyde (Sigma-Aldrich) for 10 min at room temperature; 100% methanol (chilled at −20 °C) was added, followed by incubation for 5 min at room temperature. The microfluidic system was then washed five times with ice-cold phosphate buffered saline (PBS, Thermo Fisher Scientific). For permeabilization, tumour spheroids and vessel-like tubular structure were incubated using PBS containing 0.1% Triton X-100 (Sigma-Aldrich) for 40 min at room temperature, blocked with 2% bovine serum albumin (BSA, Sigma-Aldrich) in 0.1% Tween 20 (Sigma-Aldrich) and PBS (PBST) for 45 min. The primary antibody was incubated in 1% BSA in PBST overnight at 4 °C. The spheroids were washed with PBS for 5 min and incubated with secondary antibodies (Alexa 488, Alexa 647 (Abcam, Cambridge, UK)) for 4 h at room temperature. The nuclei were counterstained with 4’,6-diamidino-2-phenylindole dihydrochloride (DAPI, Invitrogen, Carlsbad, California, USA). Primary antibodies against α-smooth muscle actin (SMA); cytokeratin; fibroblast activation protein (FAP); CD31; transforming growth factor-beta (TGFβ); collagen-I and -IV; matrix metalloproteinase (MMP)-1, -2, and -9; VE-cadherin; and VEGF were purchased from Abcam. Fluorescence images were developed using a fluorescence microscope (EVOS) and a confocal microscope (Zeiss LSM780, Carl Zeiss AG, Oberkochen, Germany). The confocal images were analyzed using ZEN Microscope Software (Carl Zeiss AG, Oberkochen, Germany).

### Scanning electron microscopy

*In vitro* MCTSs were fixed with 2.5% glutaraldehyde (Sigma-Aldrich) in deionized water (DW) for 1 h at room temperature and washed five times with DW. Secondary fixation was performed using 1% osmium tetroxide (Sigma-Aldrich) in DW for 1 h. The fixed tumour spheroids were dehydrated using a graded series of ethanol (25%, 50%, 75%, 95%, and 100%). After dehydration, the spheroids were washed three times with *tert*-butyl alcohol (Sigma-Aldrich) and frozen at −70 °C. The frozen spheroids were lyophilized until the *tert*-butyl alcohol had evaporated and were mounted on a specimen stub, coated with palladium alloy, and observed under a scanning electron microscope (AIS 1800C, SERON Tech, Korea).

### Microarray

Tumour spheroids created via co-culture with A549 and NIH3T3 cells were grown in a microfluidic system and harvested for RNA isolation at the indicated time point (Fig. 5a). Total RNA was extracted using a mirVana™ RNA Isolation Labeling Kit (Ambion, Inc, Waltham, MA). Total RNA (500 ng) was used for labeling and hybridization (Human Bead Chip V4 Microarray, Illumina, San Diego, CA), according to the manufacturer’s protocols. After scanning the bead chips using an Illumina BeadArray Reader, the microarray data were normalized using the quantile normalization method in the Linear Models for Microarray Data package in the R language environment. The expression level of each gene was log_2_ transformed before further analysis.

### Gene network and pathway analysis

Ingenuity Pathway Analysis (IPA; Ingenuity Systems, http://www.ingenuity.com) was used for gene network and pathway analysis, which was performed to identify the most significant gene sets associated with the disease process, molecular and cellular functions, and normal physiological and development conditions in genes differentially expressed between the control and co-culture, as described in the instructions from Ingenuity Systems. The significance of over-represented gene sets was estimated using a right-tailed Fisher’s exact test. Gene network analysis was performed using a global molecular network developed from information contained in the Ingenuity Knowledge Base. The features of 1,350 genes were mapped to the Ingenuity Knowledge Base. The identified gene networks were then ranked according to scores provided by IPA. These scores represent the likelihood of a set of genes being found in the networks due to random chance.

### Assessment of the anti-cancer drug response

Two types of MCTSs (with or without NIH3T3 cells) were used for comparison to assess resistance to anti-cancer drugs due to the influence of CAFs. Cell mortality in the microfluidic system was assessed by administering paclitaxel (125 nM) (Taxol, Sigma-Aldrich, USA) and gemcitabine (250 nM) (Sigma-Aldrich, USA) as anti-cancer drugs for NSCLC for 4 days. For this purpose, paclitaxel or a combination of paclitaxel + gemcitabine was diluted in the flow medium (EGM2 (Lonza, Basel, Swiss) as the endothelial cell medium, consisting of 500 ml basal medium, 5% FBS, 20 ng/ml VEGF, and 1% antibiotics) for 3D cultures after dilution with dimethyl sulfoxide (Sigma-Aldrich). The MCTSs that formed with NIH3T3 cells were compared to those formed without NIH3T3 cells. The anti-cancer drugs were administered into the microfluidic device containing MCTSs with a high circularity score (≥0.7) after tumouroid formation for 5 days. Analysis of cellular mortality to evaluate the drug was performed through fluorescence staining using the live/dead kit described earlier. Morphologies and fluorescence images of MCTSs were observed using a fluorescence microscope (EVOS; Life Technologies, USA) on days 0, 2, and 4 after treatment initiation. And, the Z-stack images were taken by confocal laser scanning microscope LSM 780 (Carl Zeiss, Jena, Germany).

### Live image capture for movie during MCTSs and vascular tube formation

The movies for tumour and vascular formation were taken using JuLI^TM^ stage (NanoEnTek, Seoul, Korea) for the live cell imaging system. Images for the movies were captured at 30-minutes intervals for 7 days.

### Statistical analysis

The circularity and fluorescence intensity of the MCTSs were determined by ImageJ Software (1.46 ver, NIH, USA). Quantitative data are presented as the mean ± standard deviation. Group differences were assessed by paired t-tests or two-way ANOVAs using GraphPad Prism 5 for Windows (GraphPad Software, Inc., GraphPad). Statistical significance was set at *p* < 0.05.

### Data Availability

All relevant data is available from the authors.

## Electronic supplementary material

Supplementary information

Supplementary Movie 1.

Supplementary Movie 2.
