# Supplementary material for: Fibroblast-associated tumour microenvironment induces vascular structure-networked tumouroid
Source: Sci Rep. 2018 Feb 5;8:2365. doi: 10.1038/s41598-018-20886-0 (PMC5799156; doi:10.1038/s41598-018-20886-0)
Supplement: Supplementary file 1 — Supplementary information [file 41598_2018_20886_MOESM1_ESM.pdf]

Scientific Reports

Supplementary Information

Fibroblast-associated tumour microenvironment induces vascular structure-networked tumouroid

Sang Woo Lee[1,2], Hyeong Seob Kwak[2], Myoung-Hee Kang[2], Yun-Yong Park[2,3] and Gi Seok Jeong[1,2,*]

[1] Biomedical Engineering Research Center, Asan Medical Center, Seoul, Korea

[2] Asan Institute for Life Sciences, Asan Medical Center, Seoul, Korea

[3]Department of Convergence Medicine, University of Ulsan College of Medicine, Seoul, Korea

***Corresponding author**: Gi Seok Jeong, PhD.

Address: Biomedical Engineering Research Center, Asan Institute for Life Sciences, Asan Medical Center, 88 Olympic-Ro, Songpa-Gu, Seoul, Korea

Postal Code: 05505

Tel: 82-2-3010-8665, 82-2- 3010-8595

E-mail: gsjeong@amc.seoul.kr

Description of Supplementary files

Description: Supplementary figures and legends

Supplementary Figure Legends

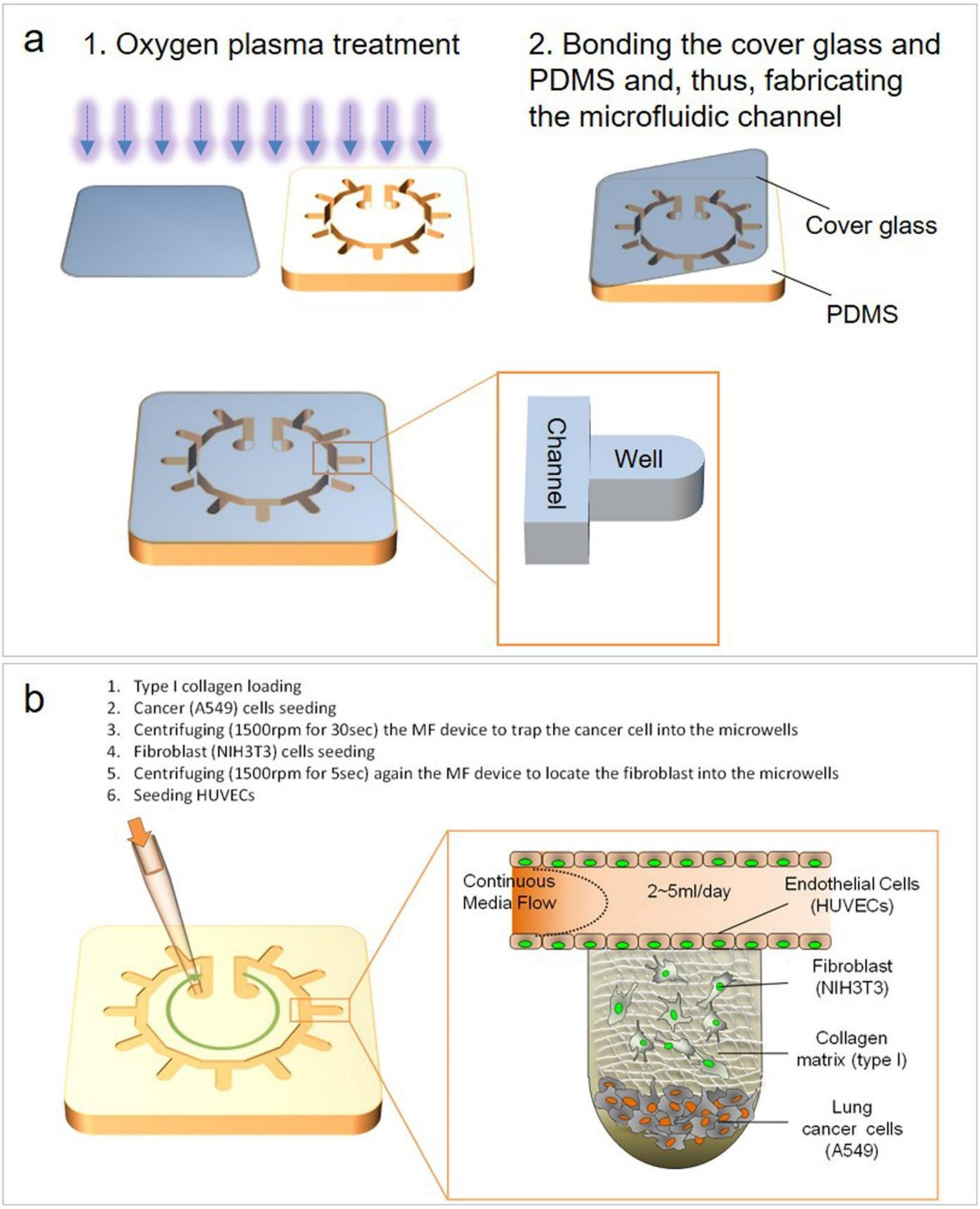

Supplementary Figure 1. Procedure for producing *in vivo*-simulating tumour microenvironment in microwells of microfluidic devices. (a) The PDMS mold and cover

glass were performed oxygen plasma treatment together. Then, PDMS and cover glass were bonded to fabricate the microfluidic channel. (b) First, the type I collagen solution was carefully loaded into the microchannel of the device. After removing the residual collagen solution from the collagen filling the microwells, cancer cells were loaded into the inlet of the device. The device was centrifuged to trap the cancer cells in the microwells, and the fibroblasts were quickly loaded into the inlet after aspiration of residual cancer cells within the microchannel. The device was centrifuged again to locate the fibroblasts into the microwells. After the device was incubated for gelation of the collagen, including cells, human umbilical vein endothelial cells (HUVECs) were loaded into the inlet.

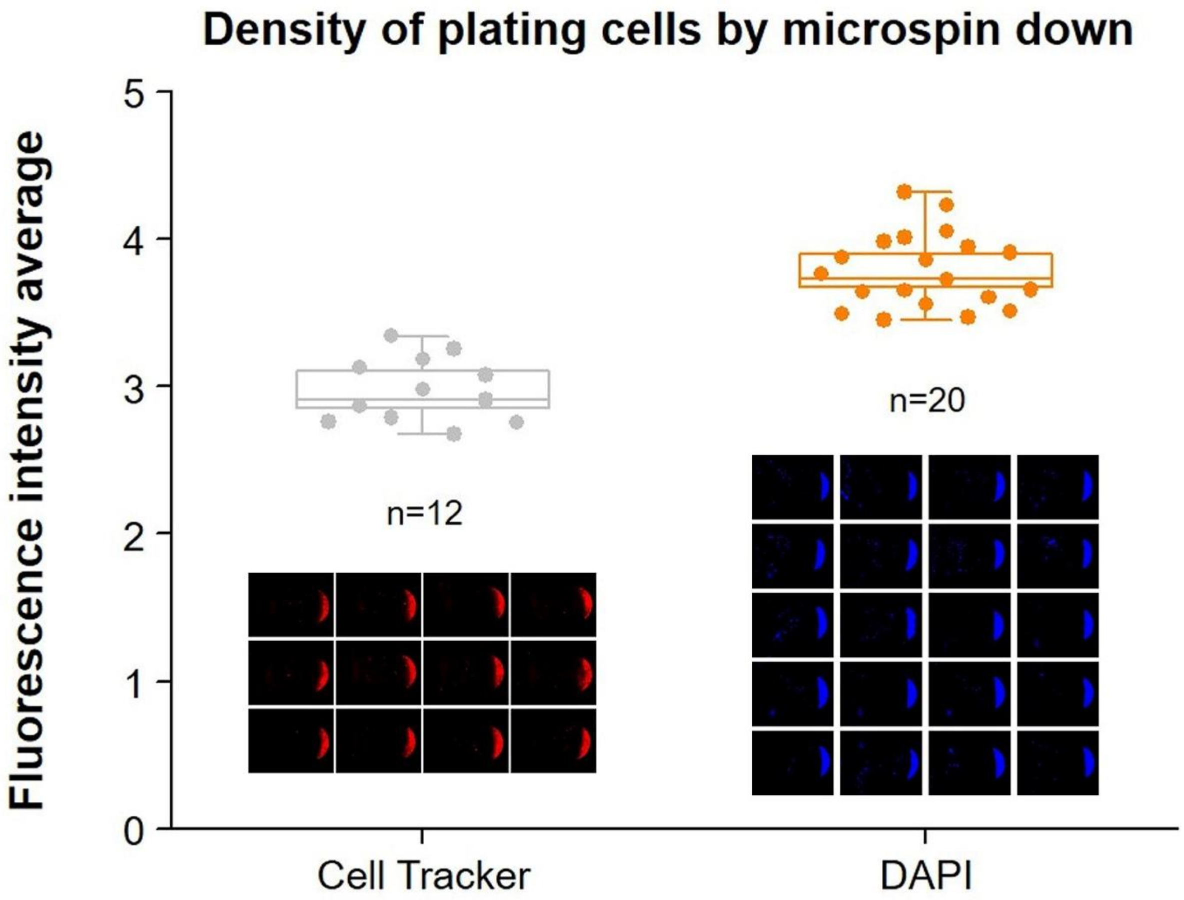

Supplementary Figure 2. Verification of the plated cell density within microwells by microspin down technique. To evaluate the similarity of plating cell density within microwells, the cells were labeled with fluorescence tracker and stained with DAPI. The

tracker labeling cells were expressed the similar fluorescence intensity within twelve microwells. Alternatively, DAPI stained cells were also confirmed the similar amount by fluorescence intensity average.

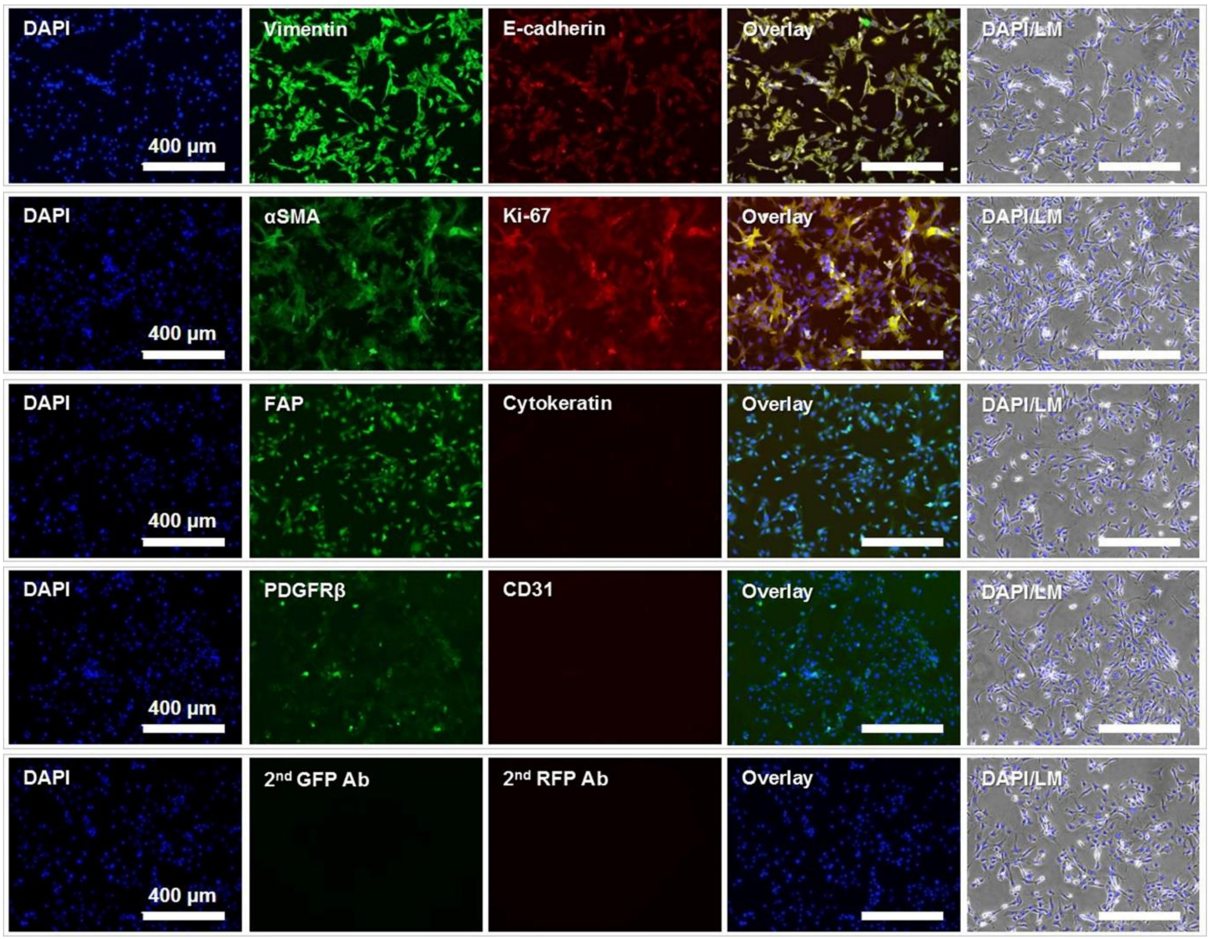

Supplementary Figure 3. Characterization of NIH3T3 cells with cancer-associated fibroblast markers. Of the epithelial–mesenchymal transition markers, vimentin for the mesenchymal lineage character was highly expressed on NIH3T3, with E-cadherin for the epithelial lineage expressed less strongly. The expression of αSMA, FAP, and PDGFRβ (typical specific positive markers of cancer-associated fibroblasts) was confirmed on cultivated NIH3T3 cells, and there was also a high level of expression of the proliferation marker Ki-67. Cytokeratin and CD31, which are negative markers, were not expressed.

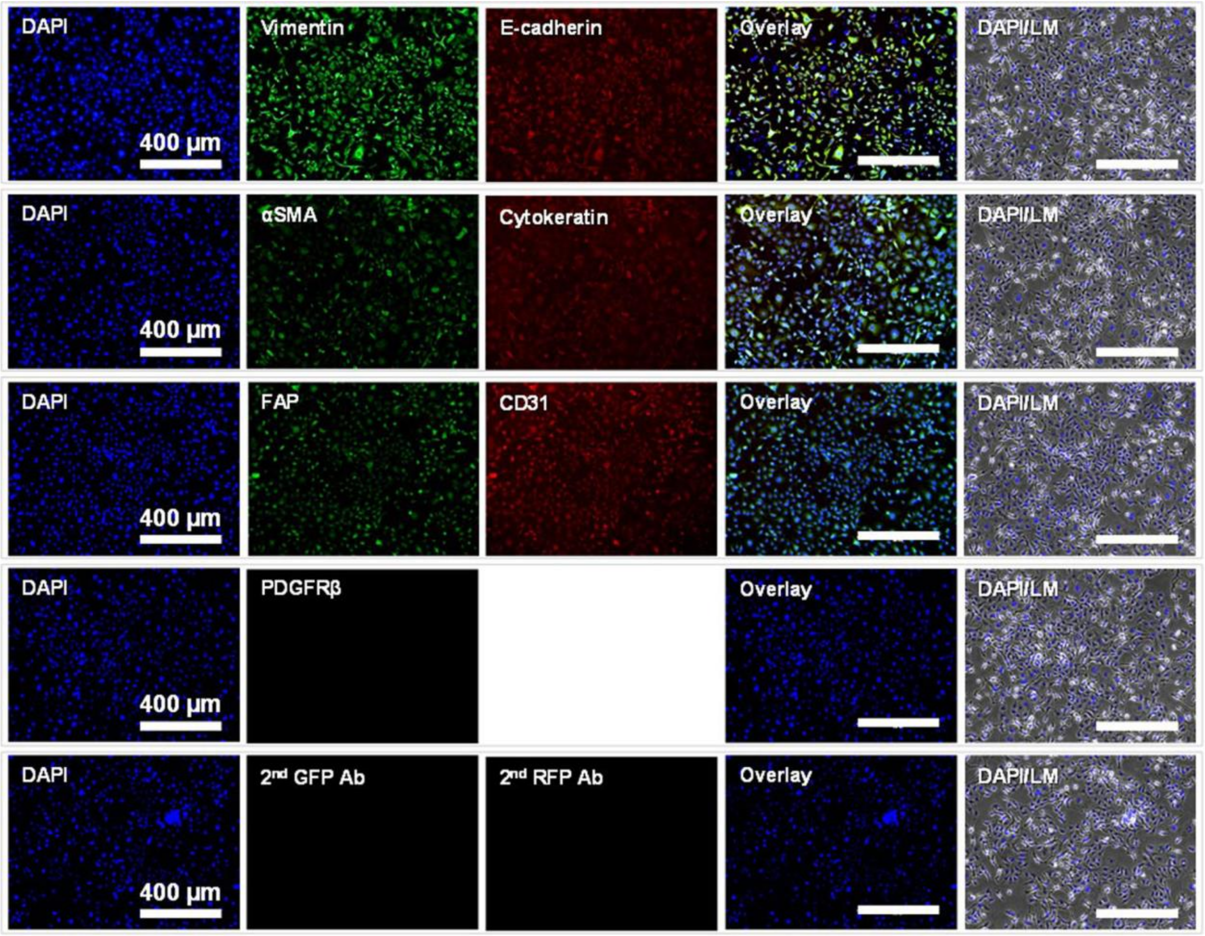

Supplementary Figure 4. Expression of cancer-associated fibroblast (CAF) markers on A549 lung cancer cells. Vimentin for the mesenchymal lineage character was highly expressed on NIH3T3. Although E-cadherin for the epithelial lineage was expressed less strongly than vimentin, it was still positively expressed. FAP and αSMA (typical specific positive markers of CAFs) were weakly expressed. PDGFRβ, another CAF marker, was not expressed. Cytokeratin and CD31, which are characteristic negative markers for CAFs, were positively expressed.

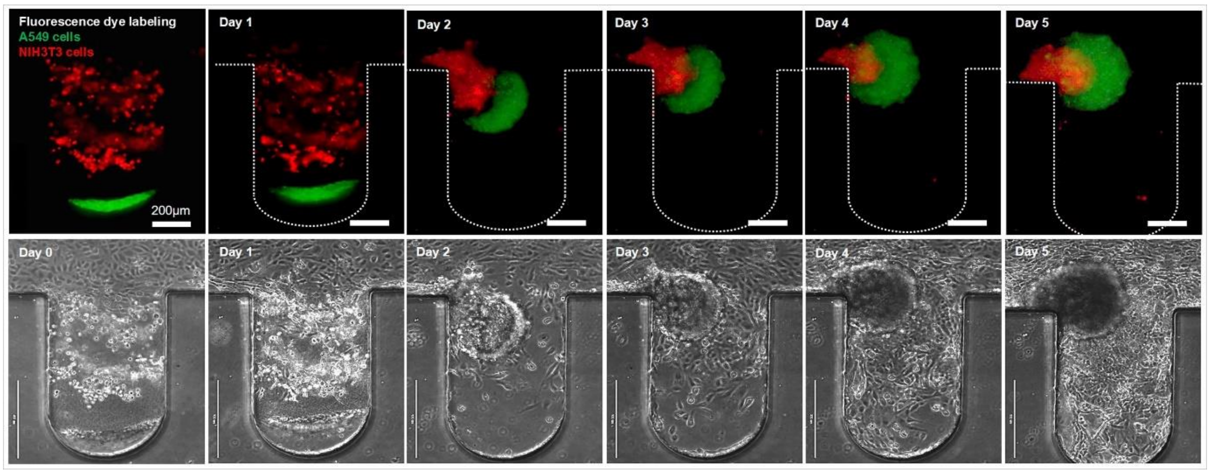

Supplementary Figure 5. Cellular complex of fluorescence-labeled A594 and NIH3T3 cells during tumour progression. Cancer and stromal cells with the collagen matrix spontaneously aggregated and formed complete spheroidal tumouroids in 2–4 days, and migrated into the channel, through which the culture medium flowed.

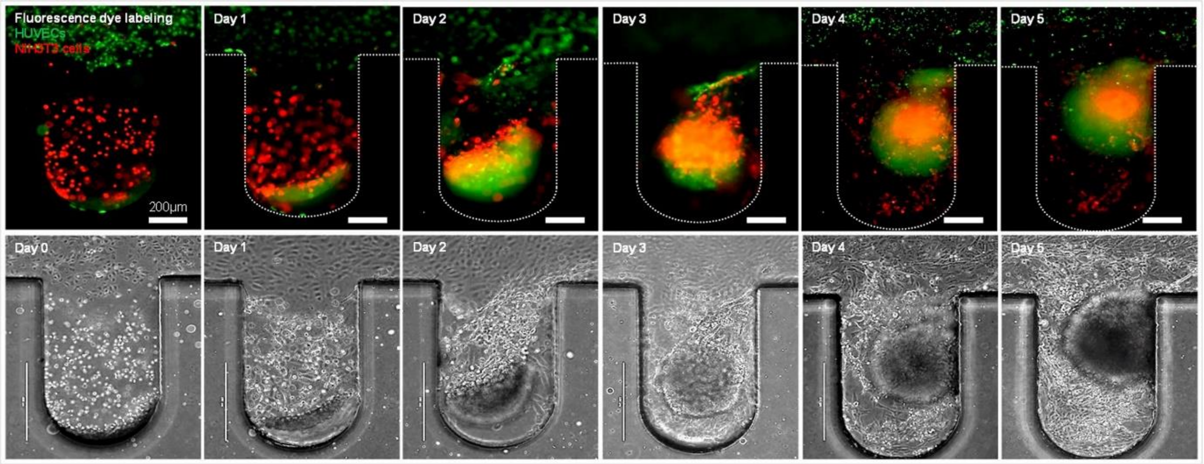

Supplementary Figure 6. Cellular complex of fluorescence-labeled human umbilical vein endothelial cells (HUVECs) and NIH3T3 cells during tumour progression. HUVECs in culture medium microchannels migrated into cancer cells within the microwell and aggregated with the *in vitro* tumouroid.

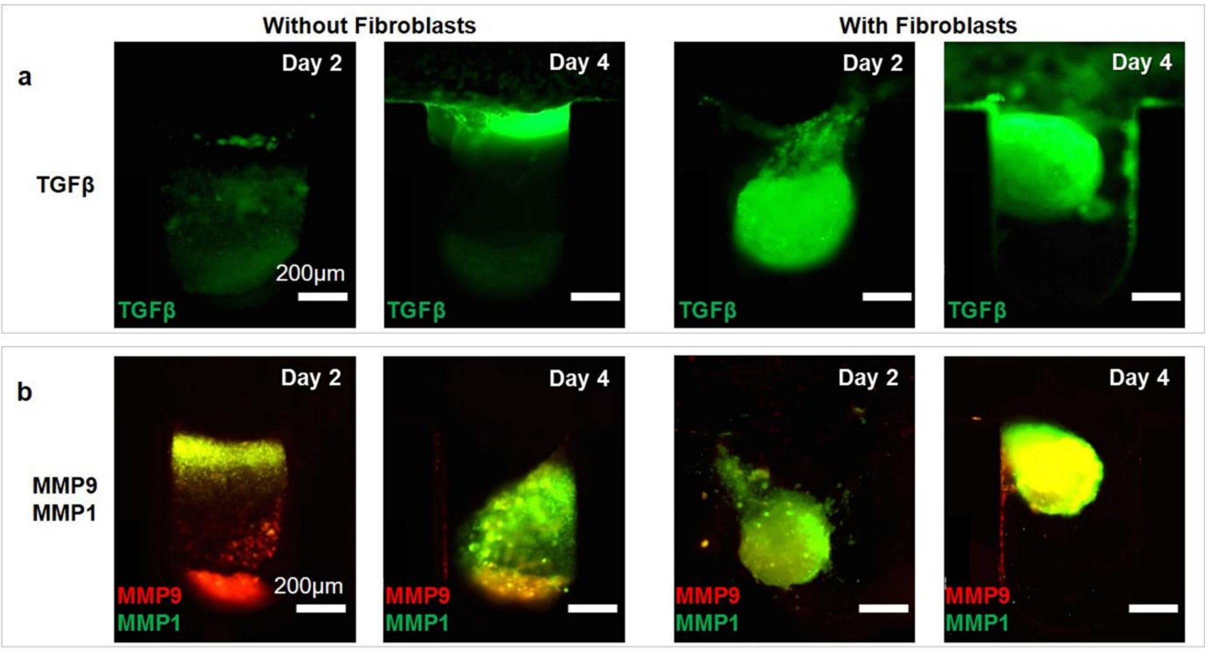

Supplementary Figure 7. TGFβ and MMP expression as the biochemical environment during *in vitro* tumour formation. (a) At the early stage of MCTS formation, TGFβ was highly expressed throughout the entire area of the *in vitro* tumouroid in the fibroblast culture model. Without fibroblasts, TGFβ was only expressed at the edge of the collagen matrix and human umbilical vein endothelial cells (HUVECs) at an early stage of the process. **(b)** MMP1 and MMP9 were highly expressed with and without fibroblast culturing. Although MMPs were highly expressed at early and late stages, there was no spheroidal formation without the fibroblast culture model. Without fibroblasts, MMP 1 and MMP9 were expressed in different areas; with the fibroblast culture model, MMP expression was relatively evenly distributed throughout the area of the *in vitro* tumouroid.

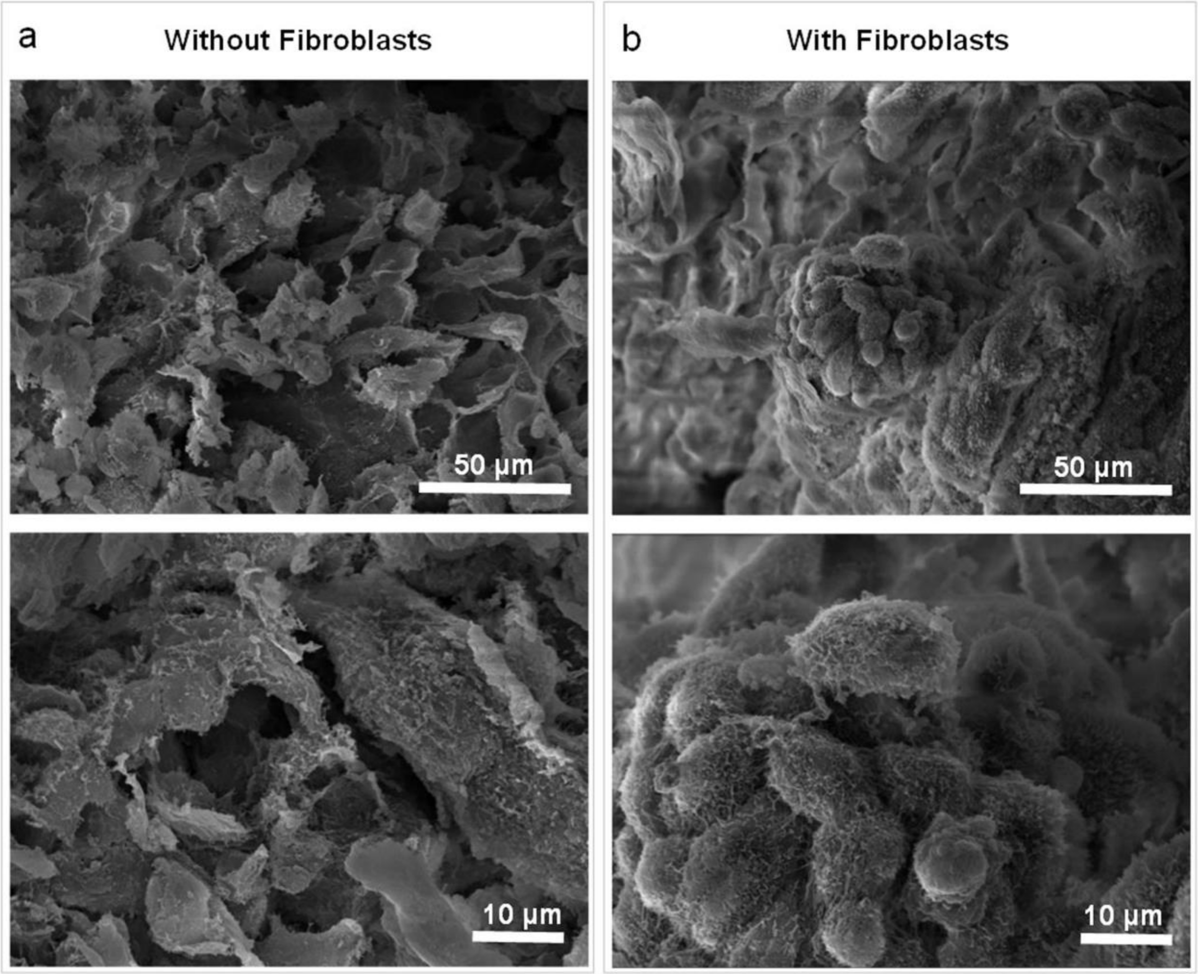

Supplementary Figure 8. Enhancement by fibroblasts of cell-to-cell interactions. Cell-to-cell interactions were observed in activated microvilli rather than in fibroblast-free tumour spheroids **(a)** or in tumours rapidly formed by fibroblasts **(b)**. According to the activation of microvilli in rapid spheroid formation by fibroblasts, cells formed a robust architectural morphology with tighter interactions than that formed without fibroblasts.

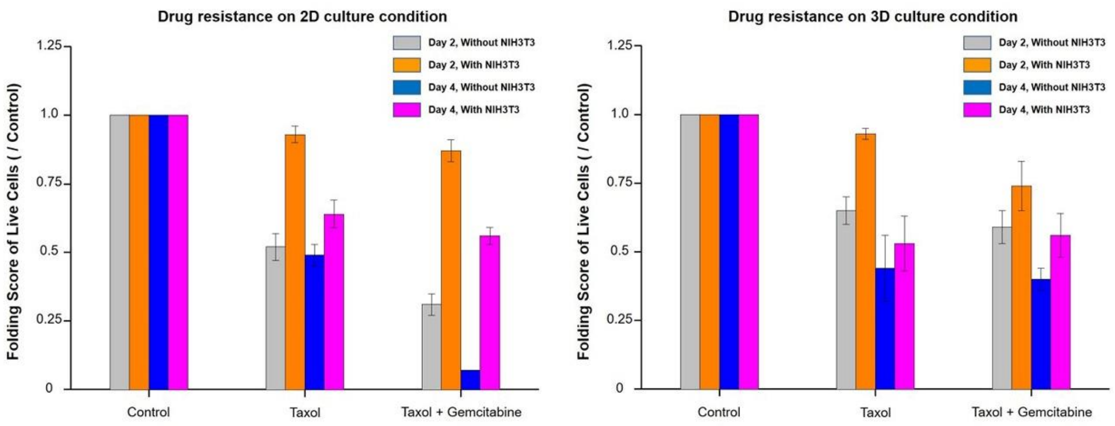

Supplementary figure 9. Comparison of drug resistance on 2D and 3D culture condition. To evaluate the drug response by the influence of NIH3T3 fibroblasts, comparative experiment was performed in 2D culture condition. The results showed that the drug resistance by NIH3T3 fibroblasts was higher through the expression of high live cell portion. However, it was lower than 3D culture condition regardless of fibroblasts.

Supplementary Movie 1. Rapid tumouroid formation by fibroblasts within microfluidic device. This movie shows formation of *in vitro* tumoroid time-lapse images in the microfluidic device. The time-lapse covers a period of about 2 days, with approximately 30 minutes elapsed time per second of movie. The time-lapse images were obtained using a real-time microscope (JuLi^{TM} stage; Nanoentek Inc., Seoul, Korea)

Supplementary Movie 2. Vasculogenesis by biophysical effect of fibroblasts within microfluidic channel after tumouroid formation. This movie shows formation of vessel-like tubular structure in the microfluidic channel. The time-lapse covers a period of about 5 days, with approximately 30 minutes elapsed time per second of movie. The time-lapse images were obtained by utilizing a real-time microscope (JuLi^{TM} stage; NanoEnTek Inc., Seoul, Korea)
